# Rethinking the economic costs of hospitalization for malaria: accounting for the comorbidities of malaria patients in western Kenya

**DOI:** 10.1186/s12936-021-03958-x

**Published:** 2021-10-30

**Authors:** Caroline Watts, Harrysone Atieli, Jason Alacapa, Ming-Chieh Lee, Guofa Zhou, Andrew Githeko, Guiyun Yan, Virginia Wiseman

**Affiliations:** 1 The Kirby Institute, University of New South Wales, Sydney, Australia; 2 Daffodil Centre, The University of Sydney, Cancer Council NSW, Sydney, Australia; 3 School of Public Health, Maseno University, Kisumu, Kenya; 4 The School of Public Health and Community Medicine, University of New South Wales, Sydney, Australia; 5 Program in Public Health, University of California, Irvine, California USA; 6 Centre for Global Health Research, Kenya Medical Research Institute, Kisumu, Kenya; 7 London School of Hygiene & Tropical Medicine, London, UK

**Keywords:** Malaria, Coinfection, Healthcare costs, Kenya, Africa

## Abstract

**Background:**

Malaria causes significant mortality and morbidity in sub-Saharan Africa, especially among children under five years of age and places a huge economic burden on individuals and health systems. While this burden has been assessed previously, few studies have explored how malaria comorbidities affect inpatient costs. This study in a malaria endemic area in Western Kenya, assessed the total treatment costs per malaria episode including comorbidities in children and adults.

**Methods:**

Total economic costs of malaria hospitalizations were calculated from a health system and societal perspective. Patient-level data were collected from patients admitted with a malaria diagnosis to a county-level hospital between June 2016 and May 2017. All treatment documented in medical records were included as health system costs. Patient and household costs included direct medical and non-medical expenses, and indirect costs due to productivity losses.

**Results:**

Of the 746 patients admitted with a malaria diagnosis, 64% were female and 36% were male. The mean age was 14 years (median 7 years). The mean length of stay was three days. The mean health system cost per patient was Kenyan Shilling (KSh) 4288 (USD 42.0) (95% confidence interval (CI) 95% CI KSh 4046–4531). The total household cost per patient was KSh 1676 (USD 16.4) (95% CI KSh 1488–1864) and consisted of: KSh 161 (USD1.6) medical costs; KSh 728 (USD 7.1) non-medical costs; and KSh 787 (USD 7.7) indirect costs. The total societal cost (health system and household costs) per patient was KSh 5964 (USD 58.4) (95% CI KSh 5534–6394). Almost a quarter of patients (24%) had a reported comorbidity. The most common malaria comorbidities were chest infections, diarrhoea, and anaemia. The inclusion of comorbidities compared to patients with-out comorbidities led to a 46% increase in societal costs (health system costs increased by 43% and patient and household costs increased by 54%).

**Conclusions:**

The economic burden of malaria is increased by comorbidities which are associated with longer hospital stays and higher medical costs to patients and the health system. Understanding the full economic burden of malaria is critical if future malaria control interventions are to protect access to care, especially by the poor.

**Supplementary Information:**

The online version contains supplementary material available at 10.1186/s12936-021-03958-x.

## Background

While there has been a worldwide decline in the number of cases and deaths over the last decade, in 2017 there were an estimated 219 million cases of malaria, and malaria control strategies continue to place a significant economic burden on many resource-constrained health systems, especially those in sub-Saharan Africa [1]. Annually in Kenya, there are an estimated 3.5 million cases diagnosed and 10,700 deaths from malaria, of which children constitute 67% [1]. Kenya has had a national strategy in place since 2004 with guidelines for vector control, malaria management in pregnancy, and strategies to improve diagnosis and treatment [1, 2]. Many endemic countries like Kenya also face increasing malaria incidence due to changing climatic and farming practices, particularly deforestation [3] as well as resistance to pyrethroid insecticides the main component found in treated bed nets and indoor household sprays [4].

Ensuring ‘affordable’ malaria treatment is a stated priority in the malaria control strategy of the Kenyan Government [5]. Notable initiatives include the abolition of user fees at primary healthcare facilities [6] and a range of malaria control programmes in areas of high malaria endemicity, including the distribution of insecticide-treated nets (ITNs), free treatment for pregnant women and children under 5 years with malaria [7], communication strategies to encourage testing [8] and the appropriate use of anti-malarial medication, intermittent preventive treatment in pregnancy (IPTp) which includes anti-malarial drugs at antenatal check-ups and a free ITNs, and the provision of indoor residual spraying (IRS) in high risk areas. Treatment for malaria is free at public hospitals and based on a recommended dosage schedule according to weight and age [9, 10]. Complicated malaria requires specialized case management depending on clinical presentation [10]. While, health insurance is available, coverage in Kenya remains low and often does not cover outpatient services such as diagnostic testing or medications [11]. As hospitals operate on cost sharing arrangements, some informal user fees and dispensary charges still apply in hospitals [6, 11].

In Kenya, malaria is the most common reason for presentation at local hospitals [12], and together with diarrhoea and pneumonia are the most common causes of death in infants under 5 years [1]. Decisions about seeking healthcare are often related to the perceived degree of morbidity risk [13, 14] and hence, it is not unusual for patients to present to hospital for treatment with more than one underlying condition [15, 16]. Some conditions such as anaemia (which is included in the national strategy for malaria control), are known to be associated with both malaria and helminth infections [17, 18]. Studies examining the co-endemicity of these infections have found that increased worm burden infection is associated with increased malaria parasitaemia [17, 18]. Other conditions, such as respiratory infections share similar symptoms to malaria, making it difficult to differentiate the cause of illness and can cause delays in seeking treatment as a respiratory infection may be expected to resolve without treatment [19]. Antibiotic use associated with diagnostic-confirmed malaria remains common [20]. Children presenting with malaria and gastrointestinal symptoms such as diarrhoea have been found to have significantly longer hospital stays than those who did not (3.13 ± 1.78 versus 2.66 ± 1.38 days) [21] and influenza has been shown to increase hospital stays by 1–3 days in settings where the typical stay was 3–5 days [22].

The economic burden of malaria inpatient and outpatient care has been well-researched, and studies on household costs have found malaria imposes significant financial burden on households [23, 24]. However, few studies in Kenya have examined the extent to which such comorbidities affect the cost of malaria treatment [25]. Ensuring affordable malaria treatment is a stated priority in Kenya’s Malaria Operational Plan [2], and health promotion messages advise patients to promptly seek confirmatory testing and use artemisinin-based combination therapy (ACT) if malaria is diagnosed [26]. The United Nations Sustainable Goals [27] note that infants and young children are more likely to be affected by malaria, and hence these families will experience greater financial burden when caregivers must take time away from paid and unpaid activities when their children are ill [20, 28]. Excluding costs of malaria comorbidities, may underestimate the actual cost of malaria-related hospitalizations [29] and mask the financial burden experienced by patients and their families [30–32]. This study assessed the comorbidities associated with malaria and their impact on hospitalizations costs, from a health system and societal perspective.

## Methods

### Study setting

This study used patient-level data from Iguhu hospital, a sub-county hospital in Kakamega county, in the malaria endemic-prone Western Kenya highlands. The hospital is funded by the Kakamega County Government Health serving a population of 17,860 people. It has 21 beds and 4 cots catering for children, women, and men. Critically ill patients are transferred to Kakamega County Teaching and Referral Hospital in Kakamega township 16 km away. The majority of the population work in small-scale subsistence farming keeping livestock, growing corn and tea or work in the informal sector [33]. Water catchments are used for agricultural purposes. Comprising both endemic and highland-epidemic prone areas, the topography, climate and farming methods favour high rates of malaria transmission [9]. A sample of children in Kakamega county found levels of parasitaemia in Kakamega to be 33%, well above the national average of 8% [34].

### Study sample

The sample comprised all infants, children, adolescents and adults admitted to Iguhu hospital with a diagnosis of malaria, associated symptoms such as fever, malaise, headache or vomiting and a laboratory confirmed *Plasmodium*-positive blood smear (or positive RDT test if laboratory testing was not available) between June 2016 and May 2017.

### Cost data collection

An ingredients-based approach [35] was used to estimate the health system and household costs of diagnosing and managing malaria hospitalizations. Patient and household costs were classified as: (1) direct costs (or out-of-pocket costs) subdivided into medical and non-medical costs; and (2) indirect costs. Direct medical costs included payments for hospital fees, medications and non-medical costs such as transport, meals and the costs of funerals for malaria related deaths. Indirect costs—including lost productive time due to travelling to hospital, being ill or providing care to a sick child—were estimated for adults over the age of 18 years using the human capital approach [36], taking a subsistence wage for an agricultural worker [37] and multiplying it by the lost time.

### Measures

To estimate resource use, detailed data were collected from hospital admission records which included: patient age, sex, village of residence, sublocation, pregnancy status, type of malaria diagnostic test conducted, type of malaria treatment, any other diagnostic tests and treatments, length of stay, discharge destination; and any fees paid by patients for their inpatient stay. Key personnel from Iguhu hospital advised on specific costs for patients, including hospital charges, transport and food, provision and stock-outs of medications in the past 12 months, and the approximate costs of these medications at local village vendors.

### Health system costs

Health system cost data were extracted from patient-level medical records and included costs associated with hospitalization (bed cost), laboratory tests, medications and related overheads (Additional file 1: Table S1). Health system costs were classified into four categories: bed day cost and a diagnostic test for malaria on admission, management of malaria, management of comorbidities, such as diarrhoea and anaemia excluding antibiotics; and antibiotics for management of infection.

### Bed cost

Inpatient cost per bed day was obtained from WHO “choosing interventions that are cost-effective (WHO-CHOICE)” framework [38]. These country-specific estimates are based on a primary level hospital with few specialties and between 30 and 200 beds and include all personnel, capital and accommodation costs excluding medications and tests. The cost per inpatient day was inflated to 2020 prices and averaged over 12 months [39]. This bed cost was multiplied by length of stay which was extracted from inpatient records.

### Laboratory test for malaria

All inpatients had confirmed malaria and the cost of microscopic diagnosis was derived from previously published estimates for Kenya [25].

### Medications for management of malaria and additional treatment

As the hospital records only listed the names of medications, dosage calculations for malaria were based on *Kenya of Government National Malaria Treatment Guidelines* [10] *for the malaria components, and for other illnesses* based on recommended dosage and duration documented in the Kenya Essential Medicines List [40], Médecins Sans Frontières (MSF) Essential Drugs [41], and MSF Clinical Guidelines [42] based on diagnosis. When recommended dosages were based on a child’s weight, approximate weights for age were calculated using the WHO weight for age charts [43] and costs were estimated based on manufacturer formulations and pack size. Dosages were independently calculated by CW and JA. When an antibiotic was recorded but an additional diagnosis was not documented (10 % cases), the most frequent diagnosis for persons of a similar age in the dataset who were also prescribed that antibiotic was used to estimate dosage.

The costs of medications and equipment were estimated using the Kenya Medical Supplies Authority (KEMSA) price list [40]. KEMSA is funded through the Ministry of Health and is responsible for the procurement and sales of essential medicines and medical supplies to government health facilities. For drugs not included on the KEMSA list, a mean cost was calculated using the Kenya Drug-Index [44]. The costs of microscopy to detect parasites in blood samples used to confirm a diagnosis of malaria were based on previously published estimates [25].

All medications listed in the hospital records and administration costs (disposable needles and syringes, IV giving sets) were included. For an additional diagnosis of dehydration, the cost for the insertion of an intravenous line and 24 h of IV fluids was included. For anaemia, where blood was required, it was assumed that one unit of packed cells was used [45], and that any oral supplements prescribed were administered for the duration of inpatient stay.

## Patient and household costs

### Direct medical costs

Direct patient costs included: registration books, laboratory test for malaria on admission and any hospital fees documented in hospital records. Children under five years of age and pregnant women were exempted from paying a registration fee and fees for malaria laboratory tests. Hospital inpatient charges to patients were included as recorded in the patient record. Any inpatient charges were assumed to be paid at discharge and deducted from the total health system cost.

Costs for medication following discharge were based on prices in the Iguhu hospital pharmacy medications price manual [46]. Hospitals set their own price for medications dispensed to discharged patients and outpatients. If the drug was not listed in the manual, the price in the KEMSA price list was doubled to reflect the price ratio in the pharmacy manual.

### Direct non-medical costs

Travel costs of patients and any accompanying persons were estimated for motorbike taxi transport. If the patient was a child under 6 years of age it was assumed that a caregiver travelled to and from the hospital with them and a meal was purchased each day. If a patient had to be transferred to Kakamega, an additional cost was estimated for a return trip. In the case of death, funeral costs were also included.

Discounting was not necessary as all costs were estimated over one year. Any costs from the published literature were converted to local currency, Kenyan Shilling (KSh) and adjusted for inflation to 2020. Changes over time in the prices of non-medical goods and services were adjusted using the Kenya consumer price index (CPI) [39], and medical costs were adjusted using the Kenyan CPI for Health [47], and then converted to USD [48].

### Statistical analysis

Patient-level data were transferred securely in a Microsoft Excel format and analysis was performed using SAS 9.4 (SAS Institute, Cary NC). Patient characteristics are described with chi-square tests used to calculate differences in proportions. Sensitivity analysis was conducted to examine the impact on household costs of an increase in hospital costs due to inflation (5%); and the impact of stockouts of antibiotics as this was reported to be a common issue by hospital personnel. If antibiotics were not available from the hospital, it was assumed they would be purchased from a local vendor. The percentage difference between the public sector (hospitals) and private sector (pharmacies) procurement prices for locally produced and imported medicines (expressed as median price ratios) [49] was used to estimate the cost increase in antibiotics if they were purchased from a local vendor. To reflect usual availability of medicines, the proportion of imported medicines (55%) and locally produced medicines (45%) were also taken into account [49]. Based on these assumptions, the price of antibiotics in the private sector was estimated to be 158% higher than in the public sector. It was assumed antibiotics were in stock 66% of the time based on the reported availability of general medicines in the public sector [49].

## Results

The sample consisted of 746 inpatients, 478 (64%) females and 268 (36%) males. The mean age for patients was 14.5 years, median 7 years (IQR 2.5–21). The median age for females was 11 years (IQR 3.4–30 years) and males 4 years (IQR: 2–9). There were 41 women admitted to hospital who were pregnant. Most patients (98%) were discharged home after the hospital stay, with 7 (1%) patient deaths and 9 (1%) hospital transfers (Table 1). Almost all individuals (98%) admitted to hospital were diagnosed with severe malaria. Intravenous artesunate was given to 98% of patients, with the remaining patients (2%) receiving quinine (intravenous or oral) or oral artesunate.

**Table 1:** Characteristics of inpatients at Iguhu hospital (n = 746)

Characteristic	n (%)
Sex	
Male	268 (36)
Female	478 (64)
Age group (years)	
≤5	322 (43)
5≤12	150 (20)
>12	274 (37)
Comorbidity	
0	567 (76)
1 or more^1^	179 (24)
Length of stay (days)	
1	36 (5)
2	189 (25)
3	323 (43)
4	100 (13)
5 or more	99 (13)
Mean length of stay (STD)	3.1 (1.3)
Outcomes	
Discharged home	720 (98)
Transferred	9 (1)
Deceased	7 (1)
*Missing*	*10*
Pregnancy status characteristics	
Pregnant	41
Mean weeks (min-max)	28 (17–35)
Age	27.5 (16–53)

^1^Includes documented treatment respiratory tract infection, dehydration and diarrhoea or dehydration and parasites, anaemia

Of the patients admitted, 179 (24%) had a documented comorbidity in addition to malaria. Younger patients (< 5 years) were more likely to have comorbidities compared to older patients (p = 0.02) (Additional file 1: Table S2). Patients with one or more comorbidities were more likely to stay longer than three days (p < 0.001). Gender was not associated with a repored comorbidity (Additional file 1: Table S1). Of the comorbidities reported within this patient group, 22% (38/171) were diarrhoea, 16 % (27/171) upper respiratory tract infection, 15% (26/171) anaemia, and 14% (24/171) pneumonia (Additional file 1: Table S3). Antibiotic use was documented for 61% (104/171) of patients and was generally consistent across all groups, ranging from 13 % of inpatients in the 5 ≤ 12 years and >12 years age groups to 16% of children up to 5 years.

Of this patient group, 614 patients (82%) incurred some direct costs. These included 167 patients who only paid registration or laboratory costs (27%) and 229 patients (37%) who paid a hospital inpatient fee of KSh 100 (USD 1). Both of these fees were paid by 209 patients (34%) and nine patients (2%) paid higher fees between KSh 200-KSh 850. (Additional file 1: Table S3). Five pregnant women paid a hospital inpatient fee.

The mean health system cost was KSh 4288 (USD 42.0) (95% confidence interval (CI) 95% CI KSh 4046–4531). Total patient and household costs averaged KSh 1676 (USD 16.4) (95% CI KSh 1488-1864) per patient, consisting of KSh 161 (USD1.6) medical and KSh 728 (USD 7.1) non-medical costs and KSh 787 (USD 7.7) indirect costs. The total societal cost (health system and household costs) was KSh 5964 (USD 58.4) (95% CI KSh 5534–6394) (Table 2).

**Table 2:** Mean health system and patient costs (KSh) related to hospitalization with confirmed malaria diagnosis

Description of cost	All patients		Patients with no comorbidity		Patients with one of more comorbidities		Hospital stay ≤ 3 days		Hospital stay > 3days	
	n=746		n=570		n=176		n=547		n=199	
	Mean cost	95 %CI	Mean cost	95 %CI	Mean cost	95 %CI	Mean cost	95 %CI	Mean cost	95 %CI
Health system cost										
Hospital admission and bed cost^1^	2650	2574-2727	2552	2474-2631	2963	2770-3156	2145	2101-2188	4052	3923-4182
Malaria management	1378	1331-1426	1324	1277-1372	1549	1427-1672	1137	1104-1170	2046	1937-2154
Additional treatment^2^	212	109-306	0	-	831	408-1253	158	54-262	363	98-628
Antibiotics	47	32-62	0	-	195	137- 254	28	20-36	100	47-152
Total health system cost	4288	4046-4531	3877	3800-4053	5583	4785-6380	3468	3393-3639	6560	6054-7166
Household costs										
Direct costs										
Medical costs										
Registration, hospital fee	118	111-125	119	111-128	113	103-122	120	113-129	111	100-123
Antibiotics	41	29-52	-	-	166	125-201	42	28-56	35	19-50
Other medication	2	1-3	-	-	8	4-12	2	1-3	3	1-6
Non-medical costs										
Transport & food cost	599	558-640	551	507-495	741	648-834	511	475-547	844	733-955
Funeral costs	129	34-225	73	0-155	308	7-609	151	30-271	70	0-207
Subtotal direct costs	*889*	*733-1044*	*743*	*608-878*	*1337*	*887-1699*	*825*	*647-1005*	*1062*	*785-1339*
Indirect costs										
Productivity loss	787	755-1044	737	703-772	945	822-960	631	601-655	1220	1149-1291
Total household costs	1676	1488-1864	1480	1311-1650	2281	1839-2502	1456	1252-1660	2283	1935-2630
Total	5964	5534-6394	5357	5061-5654	7820	6500-9139	4923	4532-5316	8843	7940-9746

^1^ Hospital admission cost includes outpatient assessment and confirmation of malaria. if patient fees were charged, these costs were deducted and included as direct costs

^2^Additional treatment relates to costs for management of comorbidities excluding costs of antibiotics

### Accounting for additional illness

**Fig. 1:**
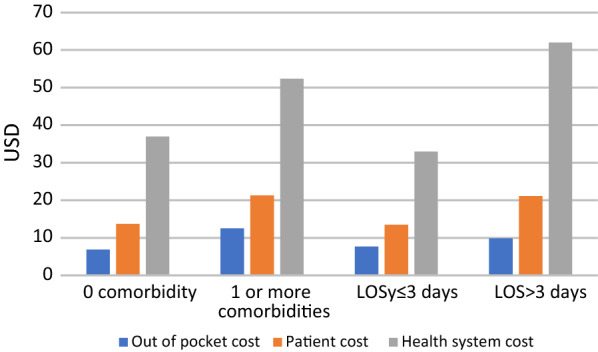
Bar chart showing difference in mean inpatient costs based on patient characteristics

Comparing patients with no comorbidities to patients experiencing one or more comorbidities, for patients with comorbidities the mean health system costs per patient increased by 43% from KSh 3877 (USD 38.0) to KSh 5538 (USD 54.3), respectively. Total mean patient and household costs increased by 54% from KSh 1480 (USD 14.5) to KSh 2281 (USD 22.4), of which direct costs increased by 80% from KSh 743 (USD 7.3) to KSh 1,337 (USD 13.1), and indirect costs due to productivity losses increased by 22% from KSh 737 (USD 7.2) to KSh 945 (USD 9.3) (Fig. 1; Table 2). Societal costs were 46% higher for admissions with at least one comorbidity in addition to malaria, compared to malaria only diagnoses (Fig. 1; Table 2).

Overall, 27% of patients were in hospital longer than the average length of stay of three days. If patients who were in hospital for three days or less, were compared with patients who stayed longer than three days, health system costs were 47% higher, increasing from KSh 3468 (USD 34.0) to KSh 6560 (USD 64.3). Total mean patient and household costs increased by 57% from KSh 1456 (USD 14.3) to KSh 2283 (USD 22.4) of which direct costs increased by 29% from KSh 825 (USD 8.1) to KSh 1062 (USD 10.4) per patient and productivity losses increased by 48% from KSh 631 (USD 6.2) to KSh 1220 (USD 12.0) per patient (Fig. 1; Table 2). Societal costs were 80% higher for patients who had longer than average stays in hospital compared to patients who stayed three days or less in hospital (Fig. 1; Table 2).

### Sensitivity analysis

Based on all patients (Table 2), a 5% increase in hospital and medicine costs increased societal costs by 4% to KSH 6186 (USD 60.6). Antibiotics availability in hospitals 66% of the time would decrease health system costs by 0.4% to KSh 4272 (USD 41.9) and increase total patient and household costs by 7% to KSh 1797 (USD 17.6), due to the 14% increase in direct medical costs which increased from KSh 889 (USD 8) to KSH 1010 (USD 9.9). Overall, the societal cost was increased by 2% to KSh 6102 (USD 59.7) (Additional file 1: Table S4). Antibiotics availability in hospitals 10%-34% of the time would increase total patient and household costs by 11 %-9 % respectively (Additional file 1: Table S4).

## Discussion

The costs associated with a visit to hospital for malaria represent a significant cost to the Kenyan health care system and to patients in malaria-endemic areas, such as Iguhu. Almost a quarter of patients admitted with malaria had at least one additional illness. While the inclusion of additional treatment provided during a hospital stay for malaria increased total health system costs the impact was greatest on household costs. Despite government policies to exempt payments for the treatment for children under 5 years and pregnant women, fees are still being incurred by these patients. Additional direct medical costs were incurred for medications and hospital inpatient fees and non-medical costs for transport and food. Transportation costs were found to be a key driver of direct patient costs, as reported in several other studies [11, 50]. The additional cost for a hospital admission of USD 7 to patients and their households is significant, given that the average income in Kenya is less than USD 3 a day.

This data aligns with other studies examining inpatient costs of malaria treatment [23, 51, 52, 53]. The mean patient cost of USD 15.5 for an average length of three days stay is similar to those reported by Kodhiambo et al. [23], an estimate of USD 10 for two days stay which was obtained from parent surveys for children with a malaria diagnosis admitted to multiple level (Level II to Level V) health facilities. The proportions of total household costs for direct medical and non-medical costs and indirect costs are quite similar between the two studies [23]. These results are also consistent with healthcare spending patterns described by Barasa et al. [11], who used the Kenya Household Expenditure and Utilization Survey and found that transport costs constituted one-third of direct inpatient costs, and also that for poorer households with limited disposable income, such costs could reduce household consumption and potentially have an impoverishing effect [11]. Household costs have also been found to increase where levels of malaria endemicity are highest, but the incremental effect of comorbidities on costs was not investigated [28]. These findings also align with the estimates of health system and direct household costs described by Sicuri et al. [52] for a hospital malaria admission without a comorbidity, however, their indirect costs are three times higher due to the inclusion of lifelong productivity losses [52], highlighting the economic impact of premature death. A study from Kenya that separated the cost of management of malaria from comorbidities, found that the addition of pneumonia increased the health system cost of hospitalization by 13% [25]. The overall health system costs for paediatric admissions in a Level IV hospital ranged from USD 47.2 to USD 75.2 in 2005, largely due to bed day costs and were much higher compared to this study [25].

There were a few limitations with this analysis. Firstly, costs may have been underestimated. Inpatient laboratory tests were not documented and therefore, not included in this cost analysis, but it can be assumed these costs would be minimal given an expectation of less complex inpatients in Level 3 hospitals. Also costs for patients who were transferred were not included as information about subsequent care or outcomes was not available. Costs may have also been missed if medications or additional medical conditions were not recorded by staff at the time of admission or discharge. Information about prior health-seeking behaviour, such as whether patients had presented on another day and been sent home, or if any other medications had been purchased prior to presenting at the hospital was not available. Secondly, shortages of general medicines in public hospitals in Kenya is not unusual [49], and was only examined in the sensitivity analysis based on general medicine shortages, rather than antibiotics which typically have lower availability [49], and hence, patient out-of-pocket costs associated with purchasing these medicine in the private retail sector may be underestimated. The use of hospital patient records, the hospital pharmacy medications and price list, and the KEMSA procurement price list meant that accurate data was obtained, avoiding recall bias which is sometimes an issue using household surveys to estimate costs [53, 54]. Thirdly, reductions in productivity may have been underestimated due to additional days not working at full capacity as it is likely that caregivers would have reduced capacity to work while also caring for children recuperating at home [24, 55]. Finally, as noted in the preceding paragraph this valuation did not include costs associated with lifelong productivity loss due to death from malaria.

Despite health financing reforms that have removed user fees in public primary health facilities and provided free maternity care [56], 82% of patients in this study admitted with malaria incurred a hospital fee which included 12% of pregnant women and around 30% of children aged under 5 years. Hospital fees were also reported in an earlier study of out-of-pocket costs for children aged under five years in Kenya [57] indicating hospital charges are still occurring in public health facilities. Out of pocket costs for patients who should be exempt from paying fees creates a risk of impoverishing families [11]. These costs can also deter people who are ill from seeking care, in turn increasing the incidence of severe disease and thus mortality [58]. Unfortunately, reasons for patient fees were not documented however hospital fees were on average slightly lower for patients with a comorbidity or with longer lengths of stay compared to the average patient, indicating some internal censoring of fees charged to patients, and that a portion of the fees may be waivered for some patients who are more ill. Regardless, a comorbidity or longer length of stay meant that households incurred higher out-of-pocket costs for transport, food, and medicines.

## Conclusions

Although the economic burden of malaria in Kenya has been assessed previously, most studies did not capture costs beyond the treatment of malaria and malaria sequelae. Comorbidities such as anaemia, diarrhoea and infections are common and increase treatment costs and length of stay, however the extent of this burden on the health system and households remains unclear. Further research is required to understand the true cost of a hospital admission for malaria in a range of malaria-endemic areas. Evidence on the aggregate inpatient costs of malaria and its comorbidities is important for designing interventions to improve access to treatment especially by the poor, and for conducting economic evaluations of these interventions.

## Supplementary Information

**Additional file 1: Table S1.** Health system costs excluding medicines. **Table S2.** Comorbidity status by patient characteristics and hospital length of stay. **Table S3.** Characteristics of patient comorbidities and hospital fee by age group (n=746). **Table S4.** Mean health system and patient costs related to hospitalisation and antibiotic stockouts with confirmedmalaria diagnosis.

## Data Availability

The datasets generated during and/or analysed during the current study are not publicly available as the data are not publicly available due to them containing information that could compromise research participant privacy/consent” but are available from the corresponding author on reasonable request.
