# Supplementary material for: Rethinking the economic costs of hospitalization for malaria: accounting for the comorbidities of malaria patients in western Kenya
Source: Malar J. 2021 Oct 30;20:429. doi: 10.1186/s12936-021-03958-x (PMC8557520; doi:10.1186/s12936-021-03958-x)
Supplement: Supplementary file 1 — Additional file 1: Table S1. Health system costs excluding medicines. Table S2. Comorbidity status by patient characteristics and hospital length of stay. Table S3. Characteristics of patient comorbidities and hospital fee by age group (n=746). Table S4. Mean health system and patient costs related to hospitalisation and antibiotic stockouts with confirmedmalaria diagnosis. [file 12936_2021_3958_MOESM1_ESM.docx]

Tables

Table S1 Costs excluding medicines

| Description | Cost (KSH) | Cost (USD) |  |
| --- | --- | --- | --- |
| Health system cost |  |  |  |
| Cost per bed day | 788 | 7.7 |  |
| Microscopy | 178 | 1.7 | ^a,b^ |
| Blood transfusion | 10458 | 102 | ^1^ |
| Intravenous line and cannula | 102 | 1.0 | ^2^ |
|  |  |  |  |
| Patient cost |  |  |  |
| Patient registration booklet (KSh 20) and malaria test (KSh 50)^a^ | 70 | 0.7 | ^a,b^ |
| Patient hospital fee | 100 | 1.0 | ^b^ |
| Transport to hospital: boda-boda | 50 | 0.5 | ^b^ |
| Cost for food for one caregiver if ≤5 years | 22 | 0.2 |  |
| Transport if referred to Kakamega hospital | 200 | 2.0 | ^b^ |
| Funeral cost | 20,000 | 12,000-35,000 | ^b^ |
|  |  |  |  |
| Lost wage for agricultural worker | 269.4 | 2.6 | ^b^ |
|  |  |  |  |

^a^ Not charged if age ≤5 years or pregnant women

^b^ Excludes 40% of patients who were not charged and 1% patients who paid >KSh100 (mean 631 (95%537-725)

Day wage applied if > 17 or

^c^ Personal communication with staff

**^1^** Matata L. A Blood Transfusion in Africa? It’s Free in Rwanda, Unaffordable in Zimbabwe [<https://globalpressjournal.com/africa/blood-transfusion-africa-free-rwanda-unaffordable-zimbabwe/>]

**^2^** Kenya Essential Medicines List 2017 [<http://www.kemsa.co.ke/salespricelist/>]

Table S2. Comorbidity status by patient characteristics and hospital length of stay

| Characteristic | No comorbidity  n=567 | One or more comorbidity  n =179 | P value |
| --- | --- | --- | --- |
|  | n (%) | n (%) |  |
| Sex |  |  |  |
| ≤3 days | 364(64) | 203 (36) |  |
| >3 days | 114 (64) | 65 (36) | 0.9 |
| Age group |  |  |  |
| 0≤5 year | 230 (71) | 92 (29) |  |
| 5≤12 | 115 (76) | 35(23) |  |
| >12 | 222 (81) | 52 (19) | 0.02 |
| Length of stay |  |  |  |
| ≤3 days | 437 (71) | 111 (21) |  |
| >3 days | 130 (66) | 68 (34) | <0.001 |

Table S3. Characteristics of patient comorbidities and hospital fee by age group (n=746)

| Admission diagnosis | Age group | | | Total ^a^ (%) |
| --- | --- | --- | --- | --- |
|  | ≤ 5 years | 5 > 12 years | ≥ 12 years |  |
|  | n (%) | n (%) | n (%) |  |
| Additional diagnosis^a^ | |  |  | |
| Diarrhoea | 23 (53) | 6 (14) | 14 (33) | 38 |
| Upper respiratory tract infection^b^ | 17 (57) | 2 (7) | 11 (37) | 30 |
| Anaemia | 16 (55) | 12(41) | 1 (4) | 29 |
| Pneumonia | 22 (88) | 1 (4) | 2 (8) | 25 |
| Meningitis | 3 (30) | 5 (50) | 2 (20) | 10 |
| Typhoid | 0 (0) | 0 (0) | 6 (100) | 6 |
| Sepsis | 2 (33) | 3 (50) | 1 (17) | 6 |
| Other^c^ | 10 (28) | 7 (19) | 29 (53) | 46 |
| Total additional diagnosis | 93 (50) | 36 (19) | 56 (30) | 185 |
|  |  |  |  |  |
| Hospital fees paid KSh |  |  |  |  |
| 0 | 96 (73) | 0 (0) | 36 (27) | 132 |
| 1-99 (0-0.9)^d^ | 0 (0) | 45 (27) | 122 (73) | 167 |
| 100 (1)^e^ | 225 (98) | 0 | 4 (2) | 229 |
| 101-200 (USD (1-2) | 0 (0) | 105 (50) | 104 (50) | 209 |
| KSh 201-KSh 900 (2-8.8) | 1 (11) | 0 (0) | 8 (89) | 9 |
| Total | 322 (43) | 150 (20) | 274 (37) | 746 |

^a^ Can have more than one co-morbidity reported

^b^ Recorded as an upper respiratory tract infection (URTI) or bronchitis

^c^ Includes HIV, asthma, helminth infection, Kwashiorkor, Tungiasis, Diabetes, Hypertension, Ulcer, antenatal management, or AB use but comorbidity not stated

^d^ Registration and laboratory test fee

^e^ Hospital stay fee

Table S4 Mean health system and patient costs (KSh) related to hospitalisation and antibiotic stockouts with confirmed malaria diagnosis.

|  | **Annual availability of antibiotics** | | | | | |
| --- | --- | --- | --- | --- | --- | --- |
|  | 10% | | 34% | | 66% | |
| **Description of cost** | Mean cost | 95%CI | Mean cost | 95%CI | Mean cost | 95%CI |
| **Health system cost** |  |  |  |  |  |  |
| Hospital admission and bed cost | 2651 | 2574-2727 | 2651 | 2574-2727 | 2651 | 2574-2727 |
| Malaria management | 1378 | 1331-1426 | 1378 | 1331-1426 | 1378 | 1331-1426 |
| Additional treatment^1^ | 212 | 109-306 | 212 | 109-306 | 212 | 109-306 |
| Antibiotics | 5 | 3-8 | 16 | 11--22 | 31 | 21-41 |
| **Total health system cost** | **4246** | **4017-4467** | **4257** | **4025-4481** | **4272** | **4035-4501** |
|  |  |  |  |  |  |  |
| **Household costs** |  |  |  |  |  |  |
| Direct costs |  |  |  |  |  |  |
| Medical costs |  |  |  |  |  |  |
| Registration, hospital fee | 217 | 111-125 | 194 | 111-125 | 162 | 111-125 |
| Antibiotics | 217 | 150-270 | 194 | 136-244 | 162 | 113-202 |
| Other medication | 2 | 1--3 | 2 | 1--3 | 2 | 1--3 |
| Non-medical costs |  |  |  |  |  |  |
| Transport & food cost | 599 | 558-640 | 599 | 558-640 | 599 | 558-640 |
| Funeral costs | 129 | 34-225 | 129 | 34-225 | 129 | 34-225 |
| ***Subtotal direct costs*** | ***1065*** | ***854-1263*** | ***1042*** | ***840-1237*** | ***1010*** | ***817-1195*** |
| Indirect costs |  |  |  |  |  |  |
| Productivity loss | 787 | 769-1144 | 787 | 755-1044 | 787 | 755-1044 |
| **Total household costs** | **1852** | **1623-2407** | **1829** | **1595-2281** | **1797** | **1572-2239** |
|  |  |  |  |  |  |  |
| **Total** | **6098** | **5640-6874** | **6086** | **5620-6762** | **6069** | **5607-6740** |
